# Impact of Vojta therapy combined with standard care on psychometric and functional parameters in patients with chronic lower back pain: a randomized controlled trial

**DOI:** 10.25122/jml-2024-0024

**Published:** 2024-05

**Authors:** Monica Elena Iosub, Sebastian Tirla, Liviu Lazar

**Affiliations:** 1 Doctoral School of Biomedical Sciences, Faculty of Medicine and Pharmacy, University of Oradea, Oradea, Romania; 2 Department of Physical Education, Sport and Physiotherapy, Faculty of Geography, Tourism and Sport, University of Oradea, Oradea, Romania; 3 Department of Psycho-Neurosciences and Recovery, Faculty of Medicine and Pharmacy, University of Oradea, Oradea, Romania

**Keywords:** low back pain, psychometric and functional parameters, Vojta therapy, classical physical therapy

## Abstract

Chronic low back pain (LBP) is very common, resulting in functional deficits and significant socio-economic burden. Non-pharmacological treatments, such as physical-psychological therapy, are frequently utilized. Vojta therapy (VT) is a type of physical therapy that effectively enhances the automatic control of body posture. This study aimed to evaluate the effects of combining VT with the usual standard of care (USC) therapy on psychometric and functional parameters in patients with chronic LBP. A total of 148 patients diagnosed with chronic LBP were recruited and randomized into two groups: LBP–VT (*n* = 82) and LBP–USC (*n* = 66). Patients were assessed for demographic characteristics, comorbid conditions, clinical findings, health status, pain symptom scales, psychometric, and functional parameters. The LBP–VT group received VT in addition to USC and electrotherapy, while the LBP–USC group received only USC. Initial Hamilton Depression Scale assessments indicated moderate depression, which improved to mild depression post-treatment. The effect of the treatment on self-esteem was significant for the LBP–VT group and moderate for the LBP–USC group. Functional parameters improved in both groups, with the LBP–VT group having significantly better results. Combining VT with standard care, electrotherapy, and massage significantly improved posture, reduced depression associated with functional deficits, and enhanced self-esteem in patients with chronic LBP.

## INTRODUCTION

Chronic low back pain (LBP) is a common condition, resulting in functional deficits and significant socio-economic burden, being the eighth leading cause of disability worldwide, often accompanied by depression and anxiety. Therapeutic management involves non-pharmacological treatment in the form of physical and psychological therapy [1,2]. LBP, defined as pain in the lower back region between the lower costal edge and above the lower gluteal folds, has been the leading cause of disability globally for the past three decades. It affects approximately 80% of the population annually, with chronic pain developing in about 20% of those affected [3,4].

LBP can be caused by multiple factors, including non-specific or mechanical issues such as discopathogenic conditions, lumbar canal stenosis, and myofascial pain. Up to 90% of chronic LBP are non-specific. Physical factors and depression increase the risk of LBP [5,6].

The classification of chronic LBP by Barrey *et al*. [7], based on lesion models, highlights three categories: non-degenerative (traumatic cause, infectious, inflammatory or tumoral processes, spondylolysis), degenerative, and unknown mechanism. This classification is essential in clinical studies to establish the selection group as clearly as possible [7].

Chronic LBP leads to physical disability, work absence, and psychological issues affecting cognitive and behavioral fields [8]. Lerman *et al*. [9] found that more than half of patients with chronic LBP exhibit depression and anxiety, which can exacerbate pain and disability. Depression is a predictor of chronicity, and its evaluation in patients with LBP is essential in establishing the treatment plan and the evolution of the patient's condition [10].

The impact of chronic LBP on an individual's work capacity, fulfillment of family responsibilities, and enjoyment of favorite activities varies depending on the severity of symptoms. Timely intervention and appropriate management can play a crucial role in alleviating pain and enhancing the patient's overall quality of life [11]. Treatment approaches generally include a combination of classic recovery therapy such as physiotherapy, pain management, and rest. In severe cases, surgical intervention may be necessary [12].

Classic recovery therapy for lower back pain combines physiotherapy with medication if needed. Physiotherapy plays a central role, focusing on reducing pain and improving both mobility and stability of the core muscles (lumbar and abdominal). Strengthening these muscles is crucial for the long-term stability of the lumbar spine [13].

Several studies suggest that deficits in motor control may underlie LBP [14]. Vojta therapy (VT), a neurophysiological approach for children and adolescents with cerebral palsy, has improved automatic body posture control by stimulating specific body activation areas [15-17]. VT addresses postural imbalances by placing patients in specific postures and applying pressure to activation zones, leading to physiological stretching of the lumbar spine and reduced mechanical stress, thereby improving function. The therapy activates muscle contractions and maintains posture until automatic control and directed phasic activity occur [18,19].

This study aimed to evaluate the evolution of psychometric and functional parameters in patients with chronic low back pain who underwent classic recovery therapy associated with VT.

## MATERIAL AND METHODS

### Study design and participants

This study included patients who visited the Băile Felix Clinical Hospital for Medical Rehabilitation in Romania between May 2020 and September 2021. The sample size was calculated based on the total number of patients attending the outpatient clinic during the study period diagnosed with chronic LBP with a degenerative cause. Several variables were considered (p - probability of occurrence of the phenomenon, 0p1, q - counter-probability, q = 1-p, t - probability factor, x - error limit, N - community volume) to determine the minimum sample size. The calculation formula used was *n* = t2 pq/(x2 + t2 pq/N). With a 95% probability (*t* = 1.96) and a limiting error of 0.1, the minimum sample size was 96.

**Figure 1:**
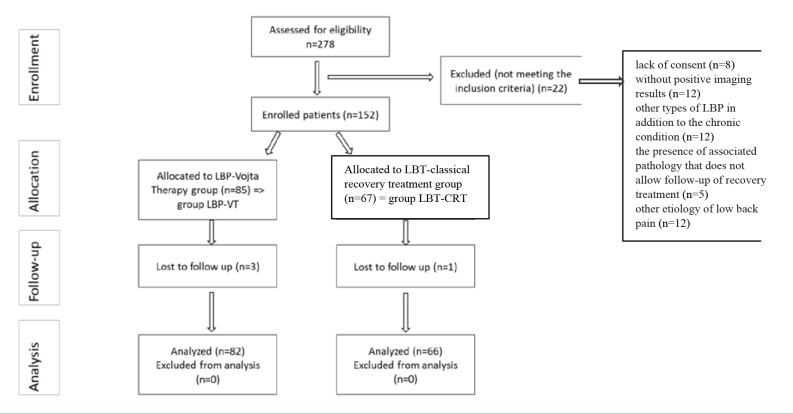
CONSORT flow diagram of the study

A total of 278 patients diagnosed with chronic LBP with a degenerative cause were recruited. Inclusion criteria included patients aged between 20 and 70 years, with chronic LBP, imaging confirmed, and willingness to participate in VT. Exclusion criteria included lack of consent, no positive imaging results, other types of LBP, associated pathologies preventing treatment, and other etiologies of low back pain (spondylolisthesis, tumors, infections). Patients were randomized into two groups based on the recommended recovery treatment (Figure 1). Simple randomization was done using sealed envelopes containing VT or the usual standard of care (USC) assignments. Patients were informed and gave written consent, understanding that group assignments were final.

Thus, they were divided into the LBP–VT group (*n* = 82), receiving VT and usual standard of care therapy, together with electrotherapy, and the LBP–USC group (*n* = 66), receiving only the usual standard of care (control group).

### Outcome measures

All patients were clinically assessed for pain using the visual analog scale (VAS), ranging from 0 to 10. Psychometric parameters were evaluated using the Hamilton Depression Rating Scale and the Morris-Rosenberg Scale (MRS). Functional parameters were evaluated with the Rolland-Morris Disability Questionnaire and the Oswestry Disability Index.

- The Hamilton Depression Rating Scale: a 21-item scale with scores ranging from 0 to 63, where higher scores indicate more severe depression. Scores range from 0 to 63, with higher scores indicating more severe depression. Interpretation: 0–7 (normal), 8–13 (mild depression), 14–18 (moderate depression), 19–22 (severe depression), 23+ (very severe depression) [20, 21].
- Morris-Rosenberg Scale (MRS): assesses self-esteem through ten statements scored from 1 to 4. Total scores range from 10 to 40, interpreted as 10–16 (low self-esteem), 17–33 (average self-esteem), and 34–40 (high self-esteem) [22].
- Roland-Morris Disability: measures functional limitations in patients with LBP, with scores ranging from 0 to 24. Higher scores indicate more severe disability [23].
- Oswestry Disability Index: assesses the impact of back pain on daily activities across ten domains, including pain intensity, personal care, lifting, walking, sitting, standing, sleeping, sex life, social life, and traveling. Scores are interpreted as 0–4 (no disability), 5–14 (mild disability), 15–24 (moderate disability), 25–34 (severe disability), and 35–50 (completely disabled) [24].

Two independent researchers carried out the assessments.

### Protocol and interventions

The study was conducted at the Băile Felix Clinical Recovery Hospital, which is known for its effective therapeutic procedures. All patients received standard care, including hydrotherapy, physical therapy, occupational therapy, massage, electrotherapy, lumbar stretching, and laser therapy.

Hydrotherapy involved 20 minutes of exercises in water at 34–36°C, focusing on various pelvic and trunk movements. Physical therapy lasted 30 minutes and used weights, elastic bands, a Bobath ball, and a fixed ladder, with exercises performed in Vojta therapy’s first position. Occupational therapy included 30 minutes of treadmill walking in the hospital, outdoor walking at home, and stationary or mobile biking.

Massage therapy was performed for 15 minutes in the prone position, using anti-inflammatory cream. Electrotherapy, lasted 12 minutes and involved applying electrodes to the lumbar spine for myorelaxation, pain relief, and vasodilation effects. Lumbar stretching and laser therapy were conducted for 10 and 8 minutes, respectively.

Patients in the LBP–VT group also received 20 minutes of VT, stimulating reflex creeping and rolling by applying pressure to specific activation zones. The treatment protocol lasted ten days, with a two-day break after five days of therapy. Initial and final assessments were conducted for all patients. The treatment was carried out for ten days for all patients, with a break of two days after five days of treatment. Each patient was given an initial assessment at the first meeting and a final assessment after the end of the last day of procedures.

### Statistical analysis

Data were processed using the JASP version 0.18.1.0. Descriptive statistics included mean values, frequency ranges, and standard deviations. The Student's t-test was used to compare means, with a significance level set at 0.05. To assess the homogeneity of the dispersion, we used Levene’s test. These tests assessed whether the variances of different groups or data sets were significantly different. If variances were not homogeneous, the Mann-Whitney U test was used. Gender distribution by pain location and time of pain onset between groups was compared using chi-square.

## RESULTS

The data distribution was consistent between the two groups regarding age, body mass index (BMI), pain location, type of pain, and onset of pain (Table 1). There were no significant differences between the two groups in terms of gender, BMI, location of pain, and the moment of its appearance (*P* > 0.05).

**Table 1:** Baseline patient demographic characteristics, comorbid conditions, clinical findings, and health status measures

Parameter		LBP–VT	LBP–USC	*P* value
Female (*n*, %)		47 (57.31)	31 (46.96)	0.817*
Average age (mean ± SD)		47.16 ± 12.54	51.12 ± 13.97	0.071**
Average height (cm) (mean ± SD)		161 ± 0.2	165 ± 3	0.685**
Average BMI (kg/m^2^)		27.48 ± 4.96	27.83 ± 5.14	0.760**
Pain location	Right (*n*, %)	25 (30.48)	21 (31.81)	0.811*
	Bilateral (*n*, %)	19 (47.50)	21 (52.50)	0.576*
Onset of pain	During sleep + upon waking (*n*, %)	5 (6.09)	2 (3.03)	0.619*
	Upon waking (*n*, %)	10 (12.18)	4 (6.06)	0.415*
	During activity (*n*, %)	45 (54.87)	44 (66.66)	0.333*

* Chi-square, **t-test.

There were no statistically significant differences between the two groups regarding the presence of De Seze, Bragard’s, and Lassegue signs at the initial and final evaluations (Table 2).

**Table 2:** Evaluation of pain scale signs

Parameters	Moment of evaluation		LBP–VT	LBP–USC	*P* value
Seze sign	Initially (mean ± SD)		26±65.00	25±62.50	0.817
	At 10 days (mean ± SD)		13±32.50	11±27.50	0.628
Bragard sign	Initially (mean ± SD)		38±95.00	35±87.50	0.817
	At 10 days (mean ± SD)		29±72.50	29±72.50	0.628
Bonnet sign	Initially (mean ± SD)		29±72.50	25±62.50	0.238
	At 10 days (mean ± SD)		12±30.00	11±27.50	0.112
Lassegue test	Initially (mean ± SD)	Cruralgia	8±20.00	3±7.50	0.107
		Ischialgia	19±47.50	24±60.00	0.265
		Sciatica	17±42.50	9±22.50	0.058
	At ten days (mean ± SD)	Cruralgia	2±5,00	2±5.00	0.062
		Ischialgia	21±52.50	22±55.00	0.824
		Sciatica	12±30.00	7±17.50	0.192

P value, independent sample t-test.

Levene's test indicated non-homogeneous dispersion for the initial depression score, Morris Rosenberg score, and initial and final Oswestry score (Table 3). The Owestry score showed significant differences between groups at the initial assessment, with group LBP–VT being more affected. However, the LBP–VT group significantly improved by the final evaluation. The mean value of the Rolland-Morris score decreased significantly in both groups.

**Table 3:** Test of Equality of Variances (Levene's test)

Parameters	F	df_1_	df_2_	*P*
Scale VAS initial points	2.599	1	146	0.109
Scale VAS final points	0.683	1	146	0.410
Hamilton scale initial points	6.526	1	146	0.012
Hamilton scale final points	0.144	1	146	0.705
Morris Rosenberg scale initial points	6.686	1	146	0.011
Morris Rosenberg scale final points	4.908	1	146	0.028
Oswestry initial score	1.415	1	104	0.237
Oswestry final score	4.046	1	104	0.047
Rolland Morris Scale initial	5.134	1	146	0.423
Rolland Morris Scale final	3.207	1	146	0.066

### Psychometric parameters

The average initial pain value for the LBP–VT group was 6.671 ± 5.831, and for the LBP–USC group, it was 7.030 ± 1.700, classified as moderate to severe pain (Table 4). There were no significant differences between the two groups (*P* = 0.629). After ten days of treatment, average pain values decreased significantly towards mild pain, with no significant differences between the groups (3.012 ± 1.991 for LBP–VT vs. 3.439 ± 1.993 for the LBP–USC group, *P* = 0.197).

Table 4 also shows no significant differences between the two groups regarding the average initial and final Hamilton scores (*P* = 0.391, respectively, *P* = 0.239). Initial scores indicated moderate depression, which improved to mild depression after treatment, with an effect size (ES) of 0.69.

**Table 4:** Comparative evolution of pain, Hamilton and Morris Rosenberg scores

Parameters	Group	Mean ± SD	*P* value
Baseline VAS score	LBP–USC	7.030 ± 1.700	0,629*
	LBP–VT	6.671 ± 5.831	
VAS scale after ten days	LBP–USC	3.439 ± 1.993	0.197*
	LBP–VT	3.012 ± 1.991	
Baseline Hamilton score	LBP–USC	17.379 ± 5.593	0.052**
	LBP–VT	15.341 ± 6.799	
Hamilton score after ten days	LBP–USC	13.803 ± 8.846	0.019*
	LBP–VT	10.390 ± 8.625	
Baseline Morris Rosenberg score	LBP–USC	25.273 ± 6.070	0.191**
	LBP–VT	26.524 ± 3.378	
Morris Rosenberg score after ten days	LBP–USC	19.606 ± 6.076	0.009**
	LBP–VT	22.098 ± 4.189	

* P value, t-test, ** P value, Mann-Whitney.

Regarding self-esteem, significant differences were observed between the two groups at revaluation (*P* = 0.025). The effect of the treatment on self-esteem was substantial in the group that followed VT treatment and usual standard of care therapy (ES=1.51) and moderate in the LBP–USC group (ES=0.69).

### Functional parameters

**Figure 2:**
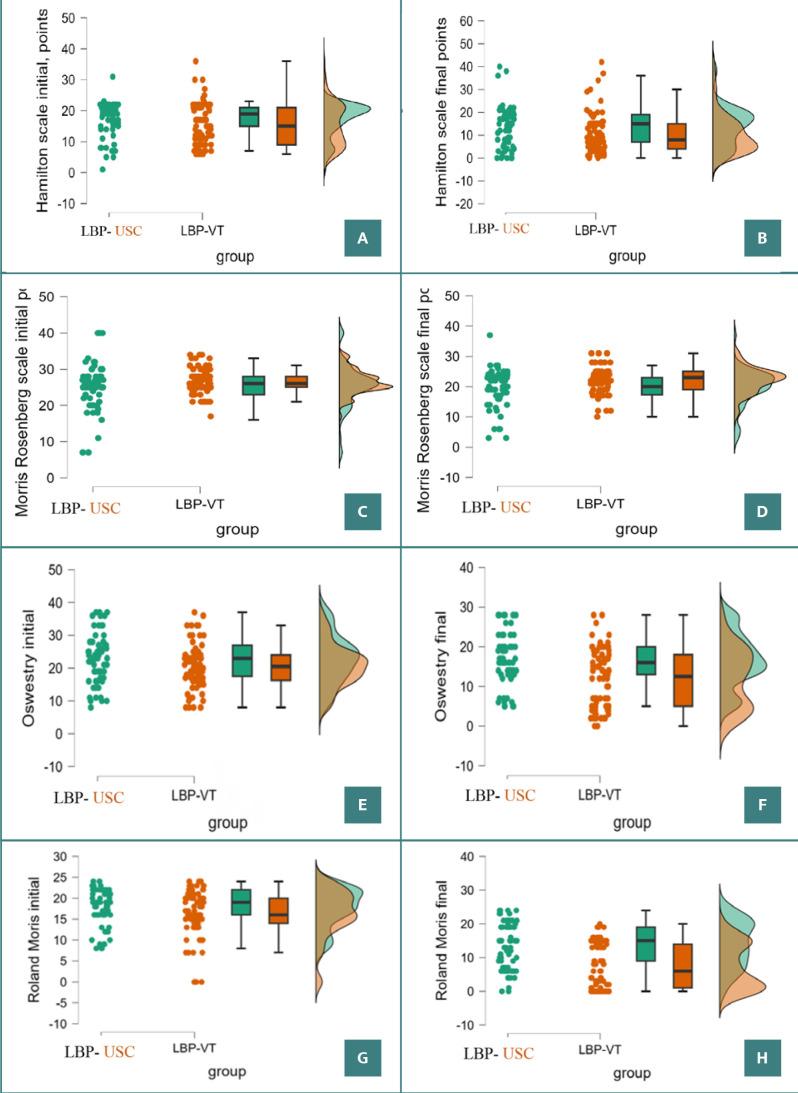
Raincloud plots illustrate the progression of disability scores for the LBP–USC and LBP–VT groups. A, Initial Hamilton scores. B, Final Hamilton scores. C, Initial Morris Rosenberg scores. D, Final Morris Rosenberg scores. E, Initial Oswestry scores. F, Final Oswestry scores. G, Initial Roland Morris scores. H, Final Roland Morris scores.

The initial average Hamilton scores indicated moderate disability in both groups: LBP–USC (17.379 ± 5.593) and LBP–VT (15.341 ± 6.799), with a *P* value of 0.052. After two weeks of treatment, the LBP–USC group had an average score of 13.803 ± 8.846, while the LBP–VT group improved to 10.390 ± 8.625 (*P* = 0.019) (Figure 2 A,B). The LBP–USC group remained in the moderate disability range, whereas the LBP–VT group moved to mild disability.

Initial Morris Rosenberg scores showed no significant differences between the groups: LBP–VT (25.273 ± 6.070) and LBP–USC (26.524 ± 3.378), with a *P* value of 0.191. However, by the second evaluation, the LBP–VT group showed significantly greater improvement (19.606 ± 6.076 vs. 22.098 ± 4.189, *P* = 0.009). The progression of disability scores is illustrated in Figure 2 C, D.

The initial average Oswestry scores also indicated moderate disability in both groups: LBP–USC (22.200 ± 8.561) and LBP–VT (20.525 ± 7.838), with a *P* value of 0.022. After two weeks of treatment, the LBP–USC group had an average score of 16.697 ± 6.679, while the LBP–VT group improved to 11.915 ± 7.399 (*P* < 0.001) (Figure 2 E, F). The LBP–USC group remained in the moderate disability range, whereas the LBP–VT group moved to mild disability.

Initial Roland Morris scores showed no significant differences between the groups: LBP–VT (16.341 ± 5.709) and LBP–USC (17.758 ± 4.661), with a *P* value of 0.106. However, by the second evaluation, the LBP–VT group showed significantly greater improvement (7.329 ± 6.566 vs. 13.894 ± 6.726, *P* < 0.001). The progression of disability scores is illustrated in Figure 2 G, H. To present a more intuitive picture of the data, including general distribution, individual trends, medians, quartiles, and outliers, we used raincloud plots (Figure 2).

**Figure 3:**
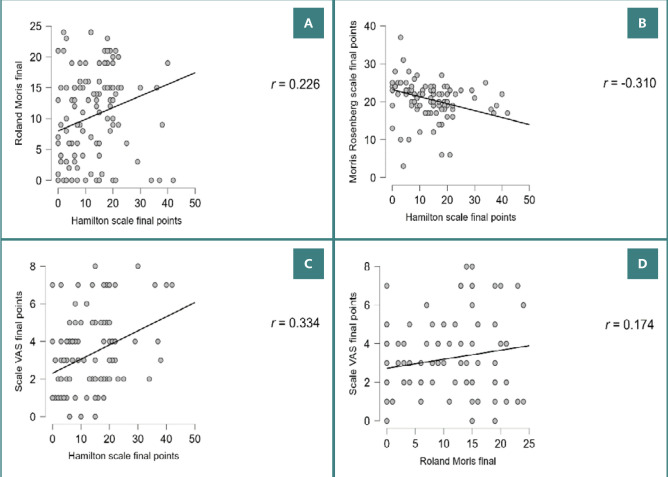
Correlation between various final scores. A, Hamilton scale final points vs. Roland Morris final points. B, Hamilton scale final points vs. Morris Rosenberg scale final points. C, Hamilton scale final points vs. Scale VAS final points. D, Roland Morris final points vs. Scale VAS final points.

Next, we assessed the correlation between the parameters for the entire study group. The study showed a moderate negative correlation between the final depression score and self-esteem (*P* <0.001, *r* = -0.310), indicating that as depression scores decreased, self-esteem increased. There was also a weak correlation between the Hamilton depression score and the Roland Moris disability score after 10 days of treatment (*P* = 0.006, *r* = 0.226). The VAS pain score showed a moderate correlation with the depression score (*P* <0.001, *r* = .0334) and a weak correlation with the disability score (*P* = 0.035, *r* = 0.174) at the end of treatment (Figure 3 A-D).

There were no negative effects following the treatment performed on any patient under observation.

## DISCUSSION

The study aimed to evaluate the benefits of VT in managing chronic low back pain. Chronic pain often limits patient activity and affects mood and self-esteem. Vojta therapy, developed by V. Vojta, stimulates specific body areas to release global motor complexes. Repeated exercises based on neuroplasticity can restore mechanisms for improved postural control and peripheral movement. Initially aimed at children with cerebral palsy, VT has proven effective in enhancing automatic control of body posture and trunk control and has been applied to adults over time [25-27].

There is a question of how VT acts on the pain mechanism. A study by Kiebzak *et al*. [28] suggests that maintaining a forced position causes discomfort and the release of stress hormones (glucocorticoids, adrenaline, and norepinephrine). Cortisol, with its proven anti-inflammatory effects, plays a role in this process. Additionally, studies have shown reduced cortisol levels in patients following regular physical therapy training, indicating a potential mechanism for pain relief [29]. While few studies support the effects of physical training on chronic pain, most have small sample sizes. The impact on psychological function varies. However, there is evidence that the quality of life of patients who follow a program supported by physical exercises improves [30].

The study assessed pain, depression, self-esteem, and disability scores (Roland Morris and Oswestry). There were no significant differences between the two groups regarding demographic characteristics and baseline clinical signs (*P* > 0.05). The Owestry score showed significant differences between groups, with the LBP–VT group improving from moderate to mild disability. The Hamilton and Morris Rosenberg scores indicated significant improvements in depression and self-esteem for the LBP–VT group compared to the LBP–USC group. The mean pain values improved in both groups, with no statistically significant differences. The mean value of pain improved in both groups regardless of the treatment followed, without statistically significant differences.

The results of a study conducted on 12 patients with LBP and radiculopathy showed that VT applied to patients with discopathy led to significantly more significant improvements in terms of pain, disability, flexibility, and radiculopathy than transcutaneous electrical nerve stimulation (TENS) application (used in the control group) [31]. Another study by Żurawski *et al*. [32] carried out on 28 patients with lumbar discopathy, underlines the effects of VT in relieving pain and normalizing posture with evolution towards the reference intervals. A meta-analysis supports the association between chronic LBP and increased risk of depression and anxiety.

The Hamilton depression score improved significantly for both groups, from moderate to mild, without significant differences. A meta-analysis of 24 studies suggests physical training (aerobic and resistance) improves mental health [33]. Regarding the Morris Rosenberg score for the self-esteem assessment, the results show a significantly higher increase in self-esteem in the group with Vojta therapy.

Two weeks after the initial assessment, the average Oswestry score for the VT group indicated mild disability, while the group that received classic recovery therapy remained in the moderate disability range. Additionally, the Roland Morris scale showed significant differences between the two groups, with the VT group demonstrating superior outcomes. These results highlight the efficacy of Vojta therapy combined with standard care in improving functional parameters compared to conventional recovery therapy alone. A study by Hamed *et al*. [34] on 40 patients with LBP compared the effects of Vojta therapy combined with TENS and standard care versus TENS and thermotherapy. The findings suggest that patients receiving Vojta therapy showed higher Oswestry scores and superior quality of life compared to those who did not follow Vojta therapy.

Additionally, a study by Fernandez *et al*. [35], involving 1,269 adult twins with an average age of 53, supports the claim that the relationship between chronic LBP and the future development of depression or anxiety symptoms is not causal. The correlation between the disability score and the self-esteem and depression scores was found to be weak, with *r* < 0.25.

### Strengths and limitations

One limitation of this study is that the Roland-Morris score does not provide detailed descriptions of different degrees of disability. Despite this, it is widely used to evaluate function in chronic LBP. Clinical improvement over time can be assessed by analyzing the evolution of questionnaire scores between two assessments and through percentage assessment. The Roland-Morris scale correlates with other physical disability measures such as the SF-36, Sickness Impact Profile, Quebec Low Back Scale, and Oswestry Questionnaire. A major strength of this study is that it is the first in Romania to track the evolution of psychometric and functional parameters in a statistically significant cohort. The incidence of depression in people with chronic LBP is 36%, and anxiety is 29%. A meta-analysis indicated that thermotherapy, ultrasound treatment, and massage without exercise yield modest pain relief, while physical training improves motor control and pain [36].

## CONCLUSION

This study supports the applicability of Vojta therapy for patients with chronic disc-pathogenic lumbar pain, in conjunction with standard care methods such as physiotherapy, electrostimulation, low and medium frequency currents, and massage. Vojta therapy improved posture to physiological levels, which in turn reduced depression associated with the functional deficits characteristic of lumbar pain and increased self-esteem. Enhanced self-confidence was correlated with less frequent pain, as evidenced by improved parameters in the Seze sign, Bragard, and Lasegue tests. These findings suggest that patient posture significantly improves with Vojta therapy.
